# Safety, Tolerability, and Pharmacokinetics of Filapixant, a Highly Selective P2X3 Receptor Antagonist, in an Ascending-Single-Dose First-in-Human Study

**DOI:** 10.3390/ph18050758

**Published:** 2025-05-20

**Authors:** Klaus Francke, Sybille Baumann, Isabella Gashaw, Stefan Klein, Beate Rohde, Oliver Zolk, Oliver M. Fischer, Christian Friedrich

**Affiliations:** 1 Bayer AG, 13353 Berlin, Germany; klaus.francke@bayer.com (K.F.); isabella.gashaw@boehringer-ingelheim.com (I.G.); stefan.klein@bayer.com (S.K.); beaterohde_23@gmx.de (B.R.); oliver-martin.fischer@bayer.com (O.M.F.); 2 CRS Clinical Research Services Berlin GmbH, 13627 Berlin, Germany; sybille.baumann@crs-group.de; 3 Institute of Clinical Pharmacology, Brandenburg Medical School, Immanuel Klinik Rüdersdorf, Seebad 82/83, Rüdersdorf bei Berlin, 15562 Rüdersdorf, Germany; oliver.zolk@mhb-fontane.de; 4 MHB—Medizinische Hochschule Brandenburg, 14770 Brandenburg, Germany

**Keywords:** dysgeusia, P2X3 receptor antagonists, pharmacokinetics, taste perception, first in human

## Abstract

**Background/Objectives:** P2X3 receptor antagonists have been suggested as a potential treatment for urogenital, respiratory and pain conditions. This first-in-human (FiH) study evaluated filapixant, a new P2X3 receptor antagonist with high receptor selectivity. It was anticipated that filapixant would cause fewer taste-related side effects compared to the unselective P2X3/P2X2/3 antagonist gefapixant and the less selective P2X3 antagonist eliapixant. This study assessed the tolerability, safety and PK of filapixant, the effect of food on PK and relative BA of a tablet vs. solution. **Methods**: This study (NCT03212586) followed a randomized, double-blind single-ascending-dose design. A total of 72 healthy male subjects received a solution (6–60 mg) or immediate-release tablets (120–1250 mg) of filapixant or corresponding placebo in fasted state. The subjects at 60 mg were re-dosed with 60 mg tablets in both fasted and fed states. The endpoints included PK parameters, dose proportionality, adverse events, and taste assessments (taste strips; dysgeusia questionnaire). **Results**: Filapixant showed dose-proportional PK with a half-life (about 10–15 h), supporting once-daily dosing. Food minimally affected PK and BA was comparable between tablet and solution. Filapixant was well tolerated; however, the number of taste side effects was unexpectedly high. Comparing the results observed across clinical filapixant studies, the threshold for such side effects seems to be well below the in vitro IC_50_ for P2X2/3. **Conclusions**: Treatment with filapixant was safe and well tolerated. Filapixant showed dose-proportional PK, bioavailability similar to that of a solution and a tablet, and a minor effect of food on PK. The number of taste side effects was unexpectedly high considering the high in vitro P2X3 receptor selectivity. Factors other than selectivity are needed to explain taste profile differences between P2X3 antagonists.

## 1. Introduction

The P2X3 receptor is a member of the P2 purinergic receptor family, which is subdivided into ligand-gated P2X receptors and G-protein-coupled P2Y receptors.

P2X3 is a non-selective cation channel activated by adenosine triphosphate (ATP), and it belongs to the P2X receptor subfamily, consisting of seven subtypes (P2X1 to P2X7). A fully functional ion channel consists of three P2X molecules, and P2X receptors can combine either as homotrimers (e.g., P2X3) or with other subtypes as heterotrimers (e.g., P2X2/3) [1,2,3]. The homomeric P2X3 receptor has been identified as a key mediator of nociception and other conditions associated with sensory-nerve-fiber overactivation [1,4,5,6,7,8]. ATP release in disorders linked to sensory-nerve-fiber overactivation occurs either directly from epithelial cells, indirectly from immune cells during inflammation, or as a result of mechanical stretch [9,10]. Activation of P2X3 receptors via ATP induces action potential in nerve fibers, which leads, e.g., to the sensation of pain, contractions of bladder or lung muscles, and ultimately to premature voiding or cough. Blocking of P2X3 receptors has therefore been considered a promising concept to treat conditions like refractory chronic cough, endometriosis, overactive bladder or neuropathic pain.

As described before, P2X3 receptor subunits can also form heterotrimers with P2X2. Among other locations, these heterotrimers are expressed in the taste buds of the tongue [11], where they play a key role in transmitting taste signals. Studies have shown that changes in taste perception occur following the nonselective blockade of both the P2X3 homomer and the P2X2/3 heteromer [2,12,13]. This is further supported by data from mice, where double-knockout models for P2X2 and P2X3 exhibited significant taste loss, whereas P2X3 single knockout did not [14,15,16].

Clinical proof of concept for refractory chronic cough, bladder pain syndrome and osteoarthritis was first established by gefapixant, a P2X3 antagonist with little selectivity over the heteromeric P2X2/3 receptor [17,18,19]. With gefapixant, a high frequency of taste-related side effects was also observed, which was explained by antagonism of the heteromeric P2X2/3 receptor. To avoid those taste-related side effects while maintaining the desired efficacy profile, there have been substantial efforts to find and develop more selective P2X3 antagonists. Here, we report the first-in-human study of the novel and selective P2X3 antagonist filapixant (BAY 1902607), which has shown effectiveness in a later study in patients with chronic cough [20]. Filapixant is chemically closely related to eliapixant, another selective P2X3 antagonist. Compared to eliapixant, the selectivity of filapixant over P2X2/3 was further improved (>100-fold vs. 20-fold; Bayer AG data on file) and thus an improved tolerability profile was also expected. This was considered necessary since eliapixant, despite showing a substantially improved tolerability profile compared to gefapixant, still led to noticeable taste disorders in up to ~20% of patients at therapeutic doses [21]. Based on preclinical data, filapixant was expected to show high bioavailability and was predicted (allometric scaling) to have a high volume of distribution (V_ss_ of 5.45 L/kg), a low-to-moderate plasma clearance (CL_plasma_ of 0.238 L/(h·kg)), and a resulting half-life suitable for once-daily dosing (t1/2 of 16 h). The available preclinical safety data supported advancements into clinical studies.

Here, we describe the first-in-human trial of filapixant (NCT03212586) that investigated its safety, tolerability, and pharmacokinetics (PK), including the relative bioavailability between different pharmaceutical formulations and the effect of food on the PK of single doses of filapixant.

## 2. Results

### 2.1. Subject Selection and Demographics

**Figure 5:**
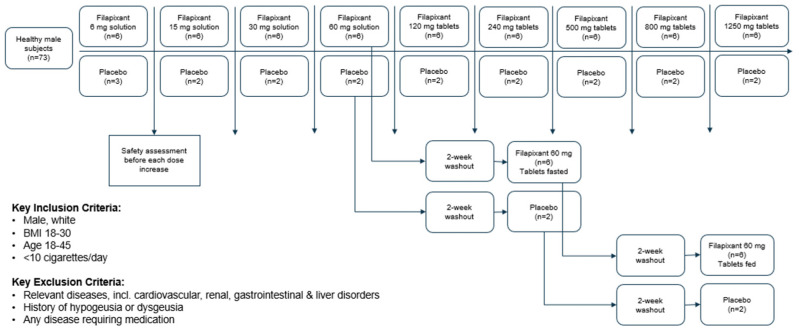
Overall study design.

A total of 72 subjects passed screening and were randomized to one of the treatments of this study. In dose group 5, a total of only seven subjects (five verum, two placebo) were randomized instead of the planned eight subjects. All 72 randomized subjects completed the treatment, including the eight subjects randomized to the 60 mg dose group that were treated three times (Figure 5) (Figure S1). All subjects were included in the safety analysis and the analysis of taste perception. All subjects treated with filapixant were included in the PK analysis. The 72 healthy subjects included in the study were of an average age of 33.5 years (range: 19 to 45 years). All subjects were male and white. Their mean weight and height at screening were 82.43 kg (standard deviation [SD]: 9.54 kg, range: 62.2 to 108.8 kg) and 181.18 cm (SD: 6.76 cm, range: 165 to 196 cm), respectively. The mean body mass index was 25.10 kg/m^2^ (SD: 2.40 kg/m^2^, range: 19.5 to 29.9 kg/m^2^).

### 2.2. PK Parameters for Single Ascending Doses Under Fasted Conditions Revealed Low Variability and Dose-Proportional Characteristics

**Figure 1:**
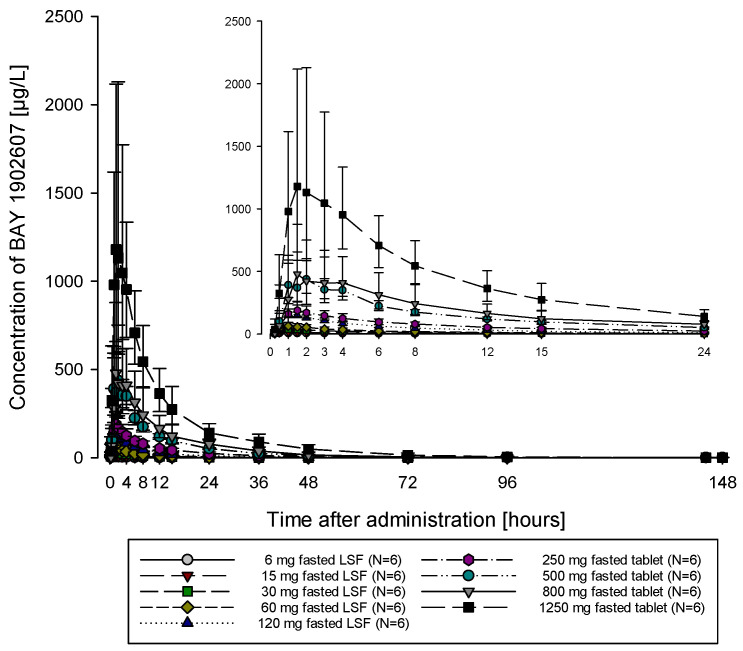

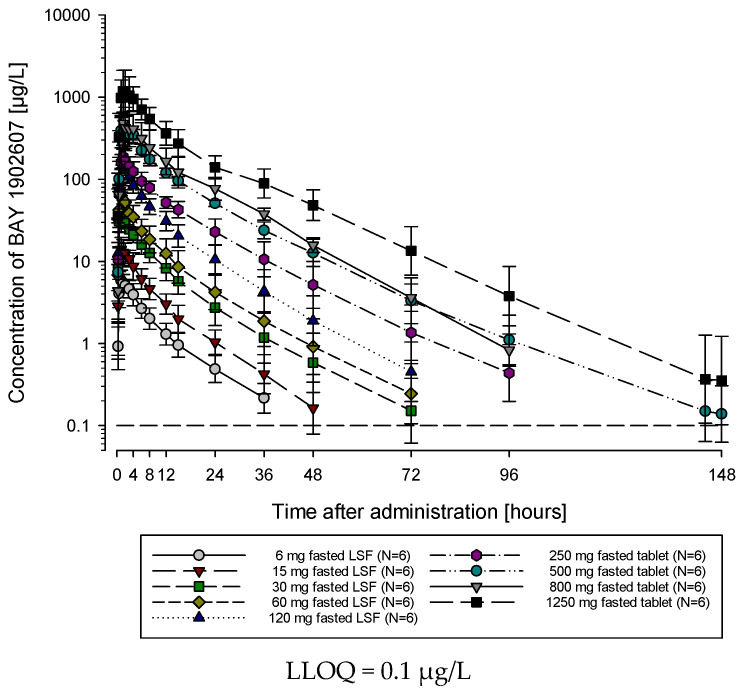
Geometric mean/SD concentration time profiles of filapixant in plasma after a single oral administration at doses between 6 and 120 mg given as a solution (liquid service formulation [LSF]) or between 250 and 1250 mg administered as a tablet, all in fasted state (linear and semi-logarithmic scale).

The subjects of all dose groups had measurable plasma concentrations above the lower limit of quantification (LLOQ) of 0.1 µg/L up to at least 36 h after dosing. Following the administration of both the solution and the tablet, filapixant was quickly absorbed, with maximum plasma concentrations observed at median values of about 1 to 1.75 h. Attainment of Cmax plasma concentration profiles was followed by an at least biphasic pattern and run roughly in parallel for all dose regimens (see Figure 1; for Gmean plasma concentration time profiles per treatment see Figures S5 and S6) with a geometric mean t_1/2_ between 9.49 and 11.7 h over the dose range 6 to 120 mg given as a solution and between 11.5 and 15.1 h after the administration of the tablet at doses of 60 mg, 250 mg, 500 mg, 800 mg and 1250 mg. Overall, no obvious dose dependency for t_1/2_ was observed, although the values tended to be lower at lower doses. This might reflect the inability to characterize the terminal phase at the lower doses as the concentration levels dropped earlier below LLOQ.

**Figure 2:**
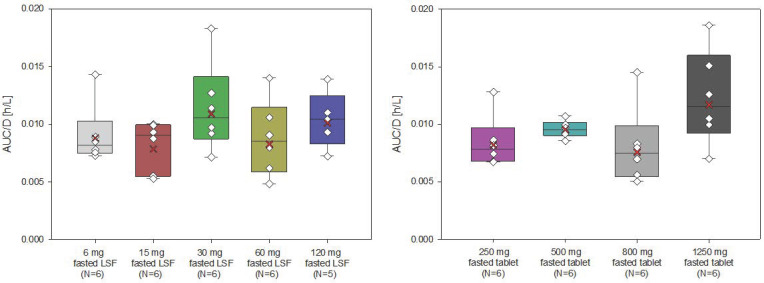
Box plot for AUC/D (h/L) of filapixant in plasma by treatment—investigation of dose proportionality (box: 25th to 75th percentile; horizontal line: median; cross: geometric mean; whiskers: 10th to 90th percentile; diamond: individual data).

**Figure 3:**
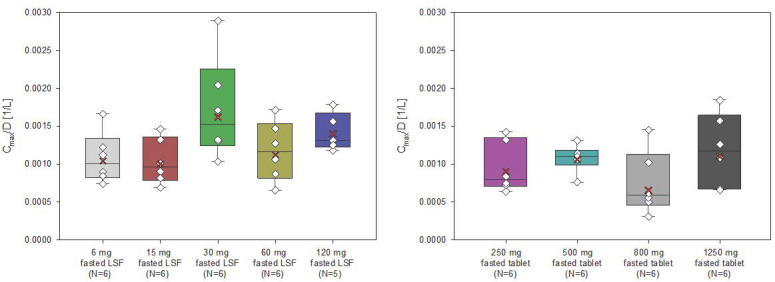
Box plot for C_max_/D (/L) of filapixant in plasma by treatment—investigation of dose proportionality (box: 25th to 75th percentile; horizontal line: median; cross: geometric mean; whiskers: 10th to 90th percentile; diamond: individual data).

The AUC and Cmax of filapixant generally increased with increasing doses and dose-normalized data showed evidence for an approximately dose-proportional increase in maximum plasma concentrations and total exposure of filapixant (Figure 2 and Figure 3). The point estimates and 90% confidence intervals (CIs) resulting from the analysis of variance (ANOVA) on dose proportionality are given in Table S3.

The least square means (LS-means) of AUC/D and Cmax/D for the different analysis groups, i.e., solution, tablet, and overall, were around zero, indicating that the increase in exposure and maximum plasma concentrations of filapixant is almost dose-proportional. In general, there is no tendency for an increase or decrease in the LS-means with increasing doses. The estimated slope parameter β of all analysis groups was between 0.95 and 1.24. Minor deviations in dose proportionality seen in the ANOVA are not considered clinically relevant.

In general, the inter-individual variability in peak plasma concentrations and total exposure of filapixant was low to moderate, and it was similar for the different dose steps and both formulations (geometric coefficients of variation [CV] between 17.2 and 58.9% for Cmax and between 7.88 and 43.4% for AUC) (Table 1).

Less than 5% of the filapixant dose was renally excreted as an unchanged substance within 24 h. Urine samples from selected dose groups of each formulation (i.e., 60 and 120 mg solution and 250 and 500 mg tablet) were analyzed first. Since no relevant excretion of filapixant in urine was detected, samples from other dose groups were not analyzed. Geometric mean renal clearance was between 4.28 and 5.38 L/h over the dose range 60 to 500 mg with no obvious dose dependency or dependency on the formulation. Additional PK parameters are given in Table S3.

### 2.3. Relative Bioavailability Was Comparable Between Solution and Tablet

The analysis of relative bioavailability was performed between the solution and tablet administered fasted at the 60 mg dose level. The administration of filapixant of both formulations resulted in similar concentration time profiles with slightly higher maximum concentrations after dosing as a solution (Figure S4).

Point estimates for the ratios of the PK parameters AUC, and Cmax as well as the exploratory 90% CIs resulting from the statistical analysis are given in Table 2.

The relative bioavailability of the tablet formulation as compared to the solution (LSF) was about 85% with respect to the overall exposure (AUC) and about 79% with respect to the maximum plasma concentration (Cmax). The result for slightly lower mean peak and total exposure after administration of the tablet was confirmed on an individual level (see stick plots in Figure S3).

### 2.4. Food Intake Had a Minor Effect on PK Properties

**Figure 4:**
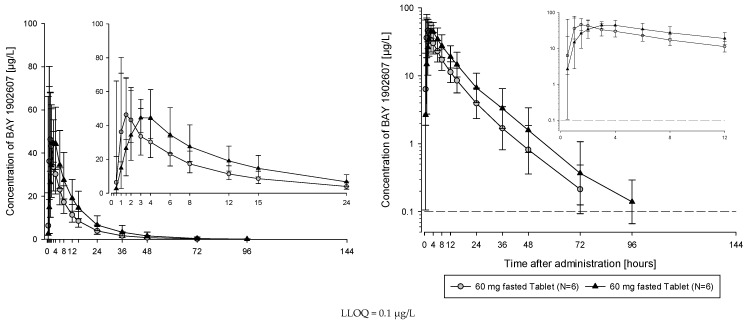
Geometric mean/SD concentration time profiles of filapixant in plasma after a single oral administration of 60 mg tablet in fasted or fed state (linear and semi-logarithmic scale).

The administration of filapixant together with a high-fat, high-caloric meal resulted in longer times to reach maximum plasma concentrations (median T_max_ + 0.75 h when compared to fasted state; 1.75 h vs. 2.25 h) and higher exposure (geometric mean AUC increased 1.5-fold when compared to fasted state), whereas the peak plasma concentrations were not affected. Mean concentration–time profiles indicate a slower absorption rate together with a prolonged absorption phase, leading to higher bioavailability (Figure 4). The increase in AUC was found to be statistically significant, while no significant effect was observed for Cmax. A similar picture is seen on an individual level (stick plots—Figure S2), where a small increase in the AUC with fed intake was consistently shown across all participants, while no consistent picture was observed for Cmax.

Point estimates for the ratios of the PK parameters AUC and Cmax, as well as the corresponding exploratory 90% CIs resulting from the statistical analysis, are given in Table 3.

In conclusion, food had only a small impact on the PK of filapixant, which can be considered clinically irrelevant considering that in a multiple-dose scenario, accumulation factors will not change as the half-life is not affected by meal intake and meal sizes are smaller, at least on average. Therefore, the filapixant tablet may be administered in fasted and fed states.

### 2.5. Safety Findings Were Consistent with the Safety Profile Known for the Class of P2X3 Antagonists

In total, 35 out of the 72 subjects (48.6%) in this study reported at least one treatment-emergent adverse event (TEAE); none of the TEAEs was serious. Out of 81 TEAEs observed in total, most TEAEs were mild in severity (78), three TEAEs were of moderate intensity, and no severe TEAE was observed. The TEAEs of moderate intensity were abdominal distension (one subject) and hypogeusia (two subjects), all seen after treatment with filapixant 1250 mg. All three TEAEs of moderate intensity were assessed as related to the study medication by the investigator. The most frequent TEAE_S_ observed with filapixant included:

- Dysgeusia (16/53 subjects, 30.2%);
- Hypogeusia (11/53 subjects, 20.8%);
- Headache (6/53 subjects, 11.3%);
- Salivary hypersecretion (3/53 subjects, 5.7%);
- Nausea (3/53 subjects, 5.7%);
- Dry mouth (2/53 subjects, 3.8%).

Overall, a higher incidence of drug-related TEAEs was seen at the higher doses, i.e., at 500, 800 and 1250 mg, which was primarily driven by taste-related events, dry mouth, salivary hypersecretion and headache. In total, 23 out of 53 subjects (43.4%) who received treatment with filapixant experienced TEAEs that were assessed as related to the study drug, and 6 out of 19 subjects who received placebo experienced TEAEs that were considered drug-related by the investigator.

All but one of the TEAEs had completely resolved by the end of this study. For one subject, a TEAE of high creatinine kinase was reported starting 3 days after study drug administration, which was assessed as related to physical activity and was partially recovered by the end of this study. Table S2 summarizes adverse events (AE) for administration of filapixant given as a solution or as a tablet in fasted or fed states.

Safety laboratory assessments and the overall pattern of vital signs and electrocardiogram (ECG) parameters revealed no clinically relevant changes after the administration of filapixant.

#### 2.5.1. Taste Strips—Relevant Effects on Taste Perception Seen with Doses of 800 mg and 1250 mg

Point estimates and 95% confidence intervals (CIs) for the difference compared to pre-dosing day in overall taste score as assessed by the taste strip test (Table 4) indicate no significant, dose-dependent influence of filapixant on taste sensation in the dose range of 6 to 500 mg. The small increase (improvement) in the taste score seen at the 500 mg dose strength might be, in light of the available dysgeusia questionnaire data and the adverse event data, rather an artefact due to the small sample size. However, after dosing of 800 mg, a trend for a reduction in taste score, and after dosing of 1250 mg filapixant, a significant reduction in taste score were observed. Considering the individual taste qualities, a notable reduction in taste score was observed for bitter and salty in subjects treated with 800 and 1250 mg filapixant, and for sour and sweet in subjects treated with 1250 mg filapixant.

#### 2.5.2. Dysgeusia Questionnaire—Relevant Effects on Taste Perception Seen at Doses of 500 mg to 1250 mg

Based on the dysgeusia questionnaire, 3/6 (50%), 5/6 (83.3%) and 6/6 (100%) subjects in the dose groups 500, 800 and 1250 mg fasted tablet, respectively, reported a dysfunction in their ability to taste after dosing of filapixant. Furthermore, 3/6 (50%), 3/6 (50.0%) and 6/6 (100%) subjects in the 500, 800 and 1250 mg groups, respectively, reported a reduction in taste sensation (i.e., hypogeusia). When additional qualification was required, distortion of the sense of taste (i.e., dysgeusia) with sweet was reported for 10 subjects at doses between 500 and 1250 mg, with salty for a total of 10 subjects at doses of 800 and 1250 mg, with hot for two subjects at 1250 mg, with sour for a total of four subjects at 800 and 1250 mg, and with bitter for two subjects at 1250 mg.

#### 2.5.3. Adverse Event Reporting—Relevant Effects on Taste Perception Seen at Doses of 500 mg to 1250 mg

TEAEs related to dysfunction in taste sensation were reported for 20/53 (37.7%) of subjects treated with filapixant and in 1/19 subjects (5.3%) of the placebo group as follows:

- Ageusia in 1 subject (1.4%) at 1250 mg;
- Dysgeusia in 17 subjects (23.6%): two subjects at 30 mg, one subject at 120 mg, one subject at placebo, two subjects each at 250 and 500 mg, five subjects at 800 mg, and four subjects at 1250 mg;
- Hypogeusia in 11 subjects (15.3%): three subjects each at 500 and 800 mg, and five subjects at 1250 mg.

Furthermore, two subjects (2.8%) of the 1250 mg dose group reported dry mouth.

In summary, the doses of 500 mg to 1250 mg showed a substantially higher rate of taste-related adverse events compared to lower doses (Table 5).

## 3. Discussion

The aim of the present first-in-human phase I study was to investigate the safety, tolerability and PK of the selective P2X3 antagonist filapixant following single ascending oral doses, including an investigation of the relative bioavailability of a filapixant solution versus tablet and the effect of a high-fat, high-calorie meal on the PK of filapixant.

Following administration of filapixant at doses of 6 to 1250 mg in a fasted state, the compound was quickly absorbed with maximum plasma concentrations observed after approximately 1 to 1.75 h. Filapixant showed approximately dose-proportional PK behavior across the entire dose range tested. The bioavailability of the tablet was about 85% based on AUC. Both the high relative bioavailability of the tablet compared to the solution and the dose-proportional PK are consistent with the high solubility and moderate-to-high permeability of filapixant observed in vitro (Bayer AG data on file). Food had a minor impact on the PK of filapixant and led to a 50% increase in the AUC, while C_max_ was not affected, which indicates that filapixant might be taken with or without food. The change in AUC was considered clinically insignificant, given the large preclinical and clinical safety window observed and under the assumption that taste-related AEs would be directly linked to drug concentrations and thus primarily driven by high C_max_ or rapid fluctuations in filapixant plasma concentrations (see later discussion on plasma drug concentration threshold). As no increase in Cmax was observed and the plasma concentration time profile with food was flatter than in a fasted state, food intake is not expected to increase the frequency of taste-related AEs.

The highest geometric mean exposure of free drug in plasma (C_max_) reached in this study was 272.6 µg/L (526 nM) at a dose of 1250 mg given as tablets (fraction unbound 19.9%). This was more than 70-fold the in vitro IC_50_ for the P2X3 homomer and about 68% of the in vitro IC_50_ for the P2X2/3 heteromer based on an in vitro fluorometric imaging plate reader (FLIPR)-based assay (IC_50_ P2X3: 7.4 nM; IC_50_ P2X2/3: 776 nM).

The half-life of filapixant was about 10 to 15 h, thus supporting once-daily dosing. For the subsequent multiple-dose escalation and proof of concept study [20,22], however, a twice-daily regimen has been chosen. This was carried out under the assumption that potential taste side effects are directly driven by drug concentrations in plasma and that a reduction in peak–trough fluctuation by twice-daily dosing would consequently allow for the maintenance of efficacious drug concentrations while avoiding taste-related side effects linked to maximum concentrations.

Overall, filapixant was well tolerated and safe at all doses administered, with no serious AEs and no relevant changes in vital signs, ECG or safety in the laboratory. The observed TEAEs were consistent with the side effect profile of a P2X3 receptor antagonist, although taste-related side effects were observed at substantial lower doses than expected based on in vitro data. At doses below 500 mg, filapixant had little impact on taste perception. However, at doses of 500 mg, 800 mg and 1250 mg, a substantial dose-dependent increase in taste-related side effects was observed, both based on spontaneous AE reporting and on the dysgeusia questionnaire. The greatest difference in spontaneous AEs to placebo was observed with the 1250 mg tablet formulation (Table 5). In addition, significant decreases were observed in the taste scores using the general taste test at 1250 mg, with a trend observable at 800 mg already. The taste test conducted at 3 h post dosing was close to the observed highest plasma concentration of filapixant and thus likely covers an effect size close to the maximum effect. In vitro studies demonstrated increasing pharmacodynamic activity with increasing concentrations of filapixant, e.g., in assays measuring intracellular calcium levels or in electrophysiological experiments. The effects on taste perception in healthy volunteers typically occurred in close temporal relationship with the highest plasma concentration of filapixant observed. However, the onset time and duration of effects were not consistently correlated to this T_max_ as the study participants also reported taste-related events until a few hours later. The duration of events was between several minutes and, in rare cases, up to approximately 24 h. These in vivo findings might be explained by certain lag times that are required until changes in taste perception are fully noticed by volunteers, and/or by a trigger like a meal, which was needed by the participants to notice taste changes. Duration, on the other hand, is dependent on sufficient blockade of P2X receptors over several hours, again coupled with variability in changes being notified by the participant. Overall, the data collected with three different methods consistently indicate a relevant effect of filapixant on taste perception in healthy volunteers with single doses above 500 mg. As mentioned before, the taste side effects observed, although expected with this mode of action, where not expected to be observed in this first-in-human study given the >100-fold selectivity for P2X3 vs. P2X3/P2X2/3 receptors of the compound (data on file, Bayer AG). However, the data are in line with the multiple-dose escalation study of filapixant, where 89% of participants reported taste-related AEs after multiple doses of 250 mg in fed state for 2 weeks, while with lower doses of 20 mg and 80 mg, only one participant in each group reported a taste-related adverse event [22]. Interestingly, the maximum concentrations reached with multiple dosing of 20 mg and 80 mg (39.6 and 133 µg/mL; Gmean) are about 13- to 4-fold lower than the concentrations achieved with the 500 mg dose in this study (533 µg/mL; Gmean), while the 250 mg multiple dose Cmax was only about 20% lower (418 µg/mL; Gmean). Also, taste-related AEs were absent after single-dose administration at Cmax of 172 µg/mL in an itraconazole drug–drug interaction study (data on file, Bayer AG). In summary, this indicates that the filapixant exposure threshold for triggering taste-related AEs is somewhere in the range of about 250 to 350 µg/mL. This corresponds to an unbound exposure of about 50 to 70 µg/mL (or 96 to 135 nM), which is surprising considering the in vitro IC_50_ for the P2X2/3 receptor being 251 to 776 nM depending on the assay used (FLIPR or MPC [manual patch clamp]; Bayer AG data on file). One explanation is that minor inhibitory effects in the range of an IC_10_ or IC_20_ are already noticed by the participant, but other factors, e.g., P2X3 homotrimer involvement in taste perception, might play a role. Please note that a recent publication has shown that most humans’ taste buds express only P2X3 homomers, which, however, needs to be confirmed in additional studies. Considering the lower selectivity of the closely related P2X3 antagonist eliapixant, which showed a substantially lower frequency of side effects at effective doses and has a very long half-life compared to filapixant, the time course of changes in receptor inhibition (either of P2X3 or P2X2/3) might also play a role. This would be in line with the shorter half-life reported for gefapixant (~7–9 hs) and its high frequency of taste-related adverse events (~60–70%) [23,24]. Unfortunately, comparisons can only be made for taste AEs as, to our knowledge, for gefapixant, no data obtained with taste strips have been reported. It can be speculated whether slow changes in receptor inhibition are unnoticed by the participants and thus the actual level of receptor inhibition becomes less relevant for compounds with longer half-lives and less peak–trough fluctuation compared to compounds with shorter half-lives and consequently more rapid changes in receptor inhibition. This could be further investigated by developing a modified release tablet of filapixant to check whether this would result in a reduced rate of side effects. On the other hand, a recent publication [25] described a second drug-binding site for P2X3 homotrimers. The interaction of filapixant with this site has not been investigated yet, nor have the binding kinetics to the P2X3 homotrimer or the P2X2/3 heterotrimer, which may also influence channel behavior and subsequent effects on efficacy, as well as taste events. Further investigations in this direction could comprise competition binding experiments, as well as manual patch clamp studies allowing for the assessment of fast on- and off-rates instead of measuring the receptor behavior at equilibrium conditions. Such studies could provide more insights for backtranslating the observed effects to the preclinical situation based on comparing the behavior of known agonists and antagonists in relation to described binding sites and their relation to potentially distinct channel functions.

Participants reporting the occurrence of taste-related AEs and describing taste impairments in the taste questionnaire largely overlapped, i.e., with both methods, relevant effects were observed from the 500 mg dose onwards. Interestingly, obvious reductions in the taste sensation in the strip test were only observed with the 800 mg dose. Whether this is a chance finding considering that AUC and C_max_ between 500 mg and 800 mg are very similar or whether this indicates a lower sensitivity of the taste strip test compared to the other methods is unclear.

Indeed, the observed differences between the different methods might be a result of methodological and sensitivity differences but could also be a result of different aspects captured by the respective test. Taste strips are expected to reflect quantitative, modality-specific changes in taste perception (hypogeusia, hypergeusia), while the questionnaire and AE reporting would also capture qualitative changes (dysgeusia).

Several limitations should be considered when interpreting the results of this study. As typical for first-in-human studies the sample size per dose group was small. This is a consequence of the FiH nature of the study, where exposure of higher subject numbers would have been unethical due to the previously unknown safety and tolerability profile. Also, the pooling of placebo subjects across different dose groups might lead to bias. On the other hand, an advantage of this study is the availability of data across several dose groups and various parameters obtained under strictly standardized conditions. The certainty of a signal observed in one dose group is largely increased if a dose–response relationship is observed and if different parameters show a consistent pattern, as was the case for the taste-related parameters in our study. Larger group sizes would have been a possibility to address high variability in some of this study’s endpoints. However, first-in-human studies, in general, have exploratory objectives. The results obtained, including variability estimates on different endpoints measured, will inform the design of subsequent clinical studies with larger samples, during which initially observed findings will be characterized further and confirmed. In addition, the benefit risk ratio between to-be-further-characterized side effects and efficacy measurements in patients needs to be established. The participants were white males only, which were chosen as no reproduction toxicity data were available at this study’s start. This was considered acceptable as no relevant differences in PK and safety were expected between males and females, even though a large proportion of the targeted indications for filapixant (chronic cough, endometriosis, overactive bladder, diabetic neuropathic pain) comprises women. Similar PK characteristics in females to those observed in male participants were confirmed in subsequent studies (Bayer AG data on file). In addition, pre-screening with the taste test might have increased the probability to detect changes in taste perception but might have led to an increased awareness and, thereby, to overestimation of the taste side effects by the study participants. However, we anticipated fewer taste perception effects due to the higher selectivity compared to eliapixant as similar conditions were employed for all healthy volunteer trials investigating filapixant and eliapixant.

## 4. Materials and Methods

### 4.1. Objectives, Study Design and Procedures

This study was conducted according to a randomized, double-blind, placebo-controlled, single-center, parallel-group, single-dose escalation design. The effect of food and the relative bioavailability between a solution (LSF = liquid service formulation) and a tablet formulation were investigated using a fixed sequence re-dosing approach. The conduct of this clinical study met all local legal and regulatory requirements and was in accordance with the ethical principles that have their origin in the Declaration of Helsinki and the International Council for Harmonisation (ICH) guideline E6: Good Clinical Practice (GCP). The subjects had to provide written, informed consent before any study-specific tests or procedures were carried out.

The primary objective of this study was to investigate the safety and tolerability of single ascending oral doses of filapixant.

Further objectives were to investigate the PK after single ascending oral doses of filapixant, to investigate the relative bioavailability of a filapixant solution compared to a tablet formulation at a dose of 60 mg, and to investigate the relative bioavailability of filapixant at a dose of 60 mg administered fasted and following a high-fat, high-caloric meal.

Filapixant was administered in fasted state in ascending single oral doses: 6 mg/15 mg/30 mg/60 mg/120 mg/250 mg/500 mg/800 mg/1250 mg. The participants in the 60 mg dose group were treated 3 times with filapixant or placebo, starting with 60 mg filapixant or placebo as a solution (fasted), followed by 60 mg filapixant or placebo as tablets (fasted), followed by 60 mg filapixant or placebo tablets administered 30 min after a high-fat, high-calorie meal (two large fried eggs, two slices of fried ham, two slices of toast and butter, pan-fried potatoes and decaffeinated coffee with milk, providing approximately 42 g protein, 67 g carbohydrate and 64 g fat (1042 kcal)). The treatment periods at the 60 mg dose were separated by a wash-out of at least 2 weeks.

In total, 73 healthy male subjects were planned to be included in this study and to receive filapixant at one of the nine sequential dose levels, i.e., 8 subjects per dose level (9 subjects at the starting dose level of 6 mg). Two subjects per dose level (three subjects for the first dose level) were allocated to placebo in a randomized, double-blind fashion to assess non-drug-related factors in the safety evaluation. Progression to the next highest dose level occurred only after careful assessment of safety and tolerability (at all dose levels) and PK (from 30 mg onwards) of the previous dose level by the principal investigator (blinded) and by the sponsor’s safety assessment group (unblinded). The study design is depicted in Figure 5.

#### 4.1.1. Materials

Filapixant was administered as a solution for doses up to 60 mg and as immediate-release tablet at doses of 60 mg and higher. The solution and the tablets used for each dose strength of filapixant were identical in size, shape, color and smell to the respective placebo formulation used in this dose step. The packaging and labeling were designed to maintain blinding to the investigator and study participants.

#### 4.1.2. Subjects

Healthy, white male subjects aged 18–45 years, with a body mass index of 18–30 kg/m^2^, who smoked < 10 cigarettes per day were allowed to participate in this study. The health status of the participants was confirmed based on a complete medical history, a physical examination, measurement of vital signs (blood pressure, heart rate, electrocardiogram), and clinical laboratory tests. Participants and their female partners of childbearing potential had to use an accepted method of contraception for the duration of this study. Subjects with relevant diseases, including cardiovascular disease, renal disorders, liver disorders, and gastrointestinal disorders, and participants with a medical history of hypogeusia or dysgeusia and existing diseases requiring medication were excluded. In addition, the participants were screened using the taste test described in Section 2.3 and the participants who could not taste at least the second highest concentration of each taste quality were excluded. The inclusion criteria on BMI, age, gender, taste and comorbidities were chosen to reduce variability in PK and PD and/or to recruit a population with low vulnerability to this first-in-human study. A full list of the exclusion criteria is provided in Table S1.

### 4.2. Assessments

#### 4.2.1. Pharmacokinetics

Blood samples for determination of filapixant in plasma were taken at the following time points: at baseline and 0.25, 0.5, 1, 1.5, 2, 3, 4, 6, 8, 12, 15, 24, 36, 48, 72, 96, 120 and 124 h after dosing. Urine for determination of filapixant was collected in the following intervals: 0–6 h, 6–12 h and 12–24 h after dosing.

Concentrations of filapixant in plasma and urine were determined using a validated high-performance liquid chromatography tandem mass spectrometry (HPLC-MS) method.

Briefly, filapixant was determined in plasma after protein precipitation with acetonitrile/ammonium acetate buffer, 2 nM, pH3; (6/1; *v*/*v*) containing an internal standard followed by filtration and subsequent evaporation to dryness with nitrogen at about 50 °C.

Afterwards, the residues were reconstituted with ammonium acetate buffer, 2 nM, pH3/acetonitrile (8/2; *v*/*v*), followed by separation employing high-pressure liquid chromatography and tandem mass spectrometric detection (LC-MS/MS).

The calibration range of the procedure was from 0.100 (LLOQ) to 100 μg/L (ULOQ). The mean inter-assay accuracy of back-calculated concentrations (except LLOQ) in calibrators ranged between 97.2% and 102% and precision was ≤5.31%. Accuracy and precision at the lowest calibrator (LLOQ) were equal to 101% and 7.29%, respectively. Quality control (QC) samples in the concentration range from 0.300 to 80.0 μg/L were determined with an accuracy of 97.8% to 101% and a precision of 3.5% to 6.1%.

Filapixant in urine was determined after addition of plasma (1:9; *v*:*v*) and thorough mixing. The subsequent handling was identical to the handling of plasma samples as described above.

The calibration range of the procedure was from 1.00 (LLOQ) to 1000 μg/L (ULOQ). The mean inter-assay accuracy of back-calculated concentrations (except LLOQ) in calibrators ranged between 98.5% and 100%, and precision was ≤5.97%. Accuracy and precision at the lowest calibrator (LLOQ) were equal to 101% and 12.5%, respectively. QC samples in the concentration range from 3.00 to 800 μg/L were determined with an accuracy of 102% to 107% and a precision of 3.5% to 6.2%.

Method validation and analysis of the study samples were performed in compliance with the pertinent guidelines on Bioanalytical Method Validation, of, e.g., the FDA (2001) and EMA (2011) [26,27].

#### 4.2.2. Safety

The safety and tolerability of filapixant were assessed by continuous recording of AEs (subjects were frequently asked for AEs in a non-leading manner) and by frequent monitoring of blood pressure, heart rate, electrocardiograms, oxygen saturation and clinical laboratory values.

To capture any potential effects of filapixant on taste perception, a general taste test was performed at baseline and 3 h after drug administration, the predicted timepoint for C_max_. The validated test (Burghart Messtechnik GmbH, Wedel, Germany) captures the ability to detect the taste qualities sweet, sour, salty and bitter [28,29]. Paper strips impregnated with the following test substances were placed on the tongues of the subjects: 0.05–0.4 g/mL sucrose (sweet); 0.05–0.3 g/mL citric acid (sour); 0.016–0.25 g/mL sodium chloride (salty); and 0.0004–0.006 g/mL quinine hydrochloride (bitter), each at 4 concentration levels. Strips with the different test substances were administered in a randomized order with increasing concentrations. The subjects were asked to identify the test quality of the respective test strips. The number of correctly identified taste strips was added up to an overall summary score, and the participants could reach a maximum score of 16 in case all strips were correctly identified. In addition, the subjects had to fill a dysgeusia questionnaire at baseline and 6 h after dosing. The dysgeusia questionnaire comprised five questions assessing the type of taste sensation (e.g., sweet, salty, sour or bitter), change in and extent of dysgeusia (e.g., no change to complete loss of taste sensation) and sensations in the mouth (e.g., dryness, burning or sour/bitter taste) [22]. Taste assessments were not carried out in the re-dosing periods of the 60 mg dose level, i.e., after the fed intake of the 60 mg tablet.

### 4.3. Data and Statistical Analyses

This was an exploratory study, and no formal statistical sample size estimation was performed. Based on general experience with other first-in-human studies, sample sizes of eight subjects (eliapixant, *n* = 6; placebo, *n* = 2) per dose level to capture the safety and tolerability of filapixant and to evaluate the relative bioavailability and a potential food interaction were considered sufficient.

All subjects who received at least one dose of the study medication were included in the safety evaluation. All subjects who received at least one dose of the study medication and who had no validity findings were included in the analysis of the taste assessments. All subjects who received filapixant and who had no validity findings affecting the PK analysis were included in the evaluation. The PK parameters were calculated by a non-compartmental analysis using the program WinNonlin version 5.3 (Pharsight Corporation, St. Louis, MO, USA), with the Automation Extension (version 2.90, Bayer Pharma AG, Wuppertal, Germany).

All statistical analyses were exploratory in nature and the variables were analyzed by descriptive statistical methods. Quantitative data were analyzed by summary statistics (mean, SD, median [range]) and frequency tables were generated for qualitative data. PK parameters are expressed as geometric mean (% coefficient of variation), apart from T_max_ and t_last_, which are expressed as median (range). Statistical evaluation was performed using the software package SAS release 9.2 or higher (SAS Institute Inc., Cary, NC, USA). To investigate dose proportionality, an explorative ANOVA, including the dose levels as an independent factor, was performed on the log-transformed PK parameters AUC/D and Cmax/D of filapixant in plasma for solution and tablet doses separately and combined. Based on these analyses, point estimates (least squares [LS]-means) and exploratory 90% confidence intervals (CIs) of the treatment effect of each dose were calculated.

In addition, the so-called power model was applied to assess dose proportionality. Here, a linear regression of log-transformed values of AUC and Cmax by log(dose) was performed. Point estimates of slope, as well as 90% CI of slope, were used to characterize the degree of dose proportionality within each analysis group. Generally, dose proportionality might have been declared over the dose range used when the CI for the slope β was contained within the range (βL, βU), where:

βL = 1 + log(0.8)/log(max(dose)/min(dose));

βU = 1 + log(1.25)/log(max(dose)/min(dose)).

The analysis of relative bioavailability was carried out for the 60 mg dose group treatment period 1 (solution administered fasted) and treatment period 2 (tablet administered fasted).

The analysis of food effect was performed for the 60 mg dose group between treatment period 2 (tablet administered fasted) and treatment period 3 (tablet administered following a high-caloric, high-fat meal).

For both comparisons, the PK parameters AUC and Cmax of filapixant in plasma were analyzed with the assumption of log-normally distributed data. The logarithms of these characteristics were analyzed in the following way: the mean value of the difference “60 mg filapixant fed tablet—60 mg filapixant fasted tablet” or “60 mg filapixant fasted tablet—60 mg filapixant fasted solution” and its 90% CI were determined assuming normal distributions. This point estimate and its CI were then exponentialized to obtain an estimate and a 90% CI for the ratio of geometric means.

## 5. Conclusions

Treatment with filapixant at single oral doses of 6 to 1250 mg was safe and well tolerated.

Filapixant had a negative impact on the perception of taste, as observed by cases of ageusia, dysgeusia or hypogeusia at doses of 500 mg and above, with the effect being consistently observed across three different evaluation methods. Taste side effects were unexpected given the high in vitro P2X3 receptor selectivity of filapixant and indicate that factors other than selectivity are important to explain the differences in P2X3 antagonists with regard to their taste-related side effect profile. Filapixant maximum plasma concentrations and total exposure increased in a generally dose-proportional manner and the observed half-life supports once-daily dosing. Food had no relevant impact on the PK of filapixant, supporting dosing in fasted and fed states.

## Figures and Tables

**Table 1:** PK parameters of filapixant in plasma after a single oral administration at doses between 6 and 120 mg given as a solution (LSF) [geometric mean/%CV (range)].

Parameter	Unit	6 mg LSF Fasted n = 6	15 mg LSF Fasted n = 6	30 mg LSF Fasted n = 6	120 mg LSF Fasted n = 5
AUC	µg⋅h/L	52.7/25.5 (43.5–85.9)	119/31.6 (79.0–160)	327/32.9 (214–549)	1210/24.3 (866–1660)
AUC/D	10^−3^⋅h/L	8.79/25.5 (7.25–14.3)	7.95/31.6 (5.27–10.7)	10.9/32.9 (7.13–18.3)	10.1/24.3 (7.21–13.9)
C_max_	µg/L	6.24/30.0 (4.44–9.96)	15.0/29.3 (10.3–21.9)	48.5/38.3 (31.0–86.8)	168/17.2 (142–214)
C_max_/D	10^−3^/L	1.04/30.0 (0.739–1.66)	0.998/29.3 (0.689–1.46)	1.62/38.3 (1.03–2.89)	1.40/17.2 (1.18–1.78)
t_max_ ^a^	h	1.01 (0.983–3.00)	1.00 (0.500–2.05)	0.992 (0.500–1.50)	1.00 (1.00–1.50)
t_1/2_	h	9.71/22.1 (7.79–14.4)	9.49/17.9 (7.06–11.4)	11.3/25.3 (7.75–14.6)	11.3/14.4 (9.18–13.6)
AE,ur	%				3.83/22.3 (2.96–5.25)
PK parameters of filapixant in plasma after a single oral administration of 60 mg given as a solution (LSF) or a tablet in fasted state or as a tablet concomitantly with food [geometric mean/%CV (range)]					
**Parameter**		**Unit**	**60 mg LSF fasted** **n = 6**	**60 mg tablet fasted** **n = 6**	**60 mg tablet fed** **n = 6**
AUC		µg⋅h/L	496/39.4 (290–841)	423/43.4 (206–649)	633/38.0 (351–945)
AUC/D		10^−3^⋅h/L	8.27/39.4 (4.83–14.0)	7.06/43.4 (3.44–10.8)	10.6/38.0 (5.86–15.7)
C_max_		µg/L	67.0/36.7 (39.3–103)	53.2/45.4 (28.2–84.7)	54.2/18.8 (45.6–75.9)
C_max_/D		10^−3^/L	1.12/36.7 (0.654–1.71)	0.886/45.4 (0.470–1.41)	0.903/18.8 (0.761–1.27)
t_max_ ^a^		h	1.26 (0.517–2.00)	1.50 (1.00–2.00)	2.25 (1.00–6.00)
t_1/2_		h	11.7/28.0 (7.04–15.8)	11.5/27.4 (7.47–15.6)	12.1/24.4 (8.23–15.9)
AE,ur		%	3.26/39.0 (1.56–5.08)		
PK parameters of filapixant in plasma after a single oral administration at doses between 250 and 1250 mg given as a tablet (geometric mean/%CV (range))					
**Parameter**	**Unit**	**250 mg tablet fasted** **n = 6**	**500 mg tablet fasted** **n = 6**	**800 mg tablet fasted** **n = 6**	**1250 mg tablet fasted** **n = 6**
AUC	µg⋅h/L	2050/24.5 (1680–3210)	4760/7.88 (4270–5360)	6050/39.0 (4000–11,600)	14,600/35.4 (8740–23,300)
AUC/D	10^−3^⋅h/L	8.20/24.5 (6.71–12.8)	9.53/7.88 (8.53–10.7)	7.56/39.0 (5.01–14.5)	11.7/35.4 (6.99–18.6)
C_max_	µg/L	225/34.5 (159–354)	532/18.1 (381–654)	523/58.9 (247–1160)	1370/44.7 (822–2300)
C_max_/D	10^−3^/L	0.901/34.5 (0.637–1.42)	1.06/18.1 (0.761–1.31)	0.654/58.9 (0.308–1.45)	1.10/44.7 (0.658–1.84)
t_max_ ^a^	h	1.25 (0.500–4.00)	1.50 (1.00–4.00)	1.50 (1.50–4.03)	1.75 (1.00–4.00)
t_1/2_	h	14.2/19.4 (11.6–18.3)	15.1/23.3 (10.6–17.8)	12.8/28.0 (10.2–20.9)	14.3/20.3 (10.1–17.4)
AE,ur	%	3.73/18.4 (3.06–4.80)	4.09/47.9 (1.39–6.71)		

^a^ Median (range).

**Table 2:** Assessment of relative bioavailability.

Ratio	Parameter	n	Geom. CV (%)	Point Estimate LS-Means	90% CI
60 mg tablet/LSF	AUC	6	8.0315	0.8532	[0.7772; 0.9366]
	Cmax	6	10.6598	0.7939	[0.7015; 0.8984]

**Table 3:** Assessment of food effect on the PK of filapixant.

Ratio	Parameter	n	Geom. CV (%)	Point Estimate LS-Means	90% CI
60 mg tablet fed/fasted	AUC	6	7.0103	1.4953	[1.3784; 1.6222]
	C_max_	6	21.5947	1.0194	[0.7952; 1.3068]

**Table 4:** Overall taste strip scores as differences compared to pre-dosing day (negative differences indicate taste impairment.

Treatment	Time After Dosing with Filapixant	Number of Subjects	Point Estimate (Mean)	95% CI
6 mg fasted LSF	0D03H00M	6	0.50000	[−0.60066; 1.60066]
15 mg fasted LSF	0D03H00M	6	1.00000	[−0.62578; 2.62578]
30 mg fasted LSF	0D03H00M	6	0.33333	[−0.75052; 1.41719]
60 mg fasted LSF	0D03H00M	6	0.66667	[−0.60426; 1.93760]
60 mg fasted tablet	0D03H00M	6	0.16667	[−1.06017; 1.39350]
120 mg fasted LSF	0D03H00M	5	−1.00000	[−3.91196; 1.91196]
250 mg fasted tablet	0D03H00M	6	0.33333	[−1.24664; 1.91331]
500 mg fasted tablet	0D03H00M	6	1.83333	[0.28861; 3.37806]
800 mg fasted tablet	0D03H00M	6	−1.33333	[−3.17109; 0.50443]
1250 mg fasted tablet	0D03H00M	6	−4.83333	[−7.35344; −2.31323]
Placebo fasted solution	0D03H00M	11	1.00000	[−0.20177; 2.20177]
Placebo fasted tablet	0D03H00M	10	0.60000	[−1.85964; 3.05964]

**Table 5:** Number of subjects with taste-related AEs per dose group.

Treatment	Number of Subjects	Hypogeusia	Dysgeusia	Ageusia	Subjects with Taste-related AEs Total
6 mg	6				
15 mg	6				
30 mg	6		2		2 (33%)
60 mg (solution)	6				
60 mg (tablet fasted) 60 mg (tablet fed)	6				
120 mg	5		1		1 (17%)
250 mg	6		2		2 (33%)
500 mg	6	3	2		4 (67%)
800 mg	6	3	5		5 (84%)
1250 mg	6	5	4	1	6 (100%)
Placebo	21		1		1 (5%)

## Data Availability

Availability of the data underlying this publication will be determined according to Bayer’s commitment to the European Federation of Pharmaceutical Industries and Associations and Pharmaceutical Research and Manufacturers of America principles for responsible clinical trial data sharing, pertaining to scope, time point and process of data access. Bayer commits to sharing, upon request from qualified scientific and medical researchers, patient-level clinical trial data, study-level clinical trial data and protocols from clinical trials in patients for medicines and indications approved in the USA and European Union as necessary for carrying out legitimate research. This commitment applies to data on new medicines and indications that have been approved by the European Union and US regulatory agencies on or after 1 January 2014. Interested researchers can use https://www.clinicalstudydatarequest.com/ to request access to anonymized patient-level data and supporting documents from clinical studies to conduct further research that can help advance medical science or improve patient care. Information on the Bayer criteria for listing studies and other relevant information is provided in the study sponsors’ section of the portal. Data access will be granted to anonymized patient-level data, protocols and clinical study reports after approval by an independent scientific review panel. Bayer is not involved in the decisions made by the independent review panel. Bayer will take all necessary measures to ensure that patient privacy is safeguarded.

## Supplementary Materials

The following supporting information can be downloaded at: https://www.mdpi.com/article/10.3390/ph18050758/s1, Figure S1: Subject disposition; Figure S2: Stick plot for AUC (µg∙h/L) and C_max_ (µg/L) of filapixant in plasma by treatment—investigation of food effect (logarithmic scale); Figure S3: Stick plots for AUC (µg∙h/L) and Cmax (µg/L) of filapixant in plasma by treatment—investigation of relative bioavailability (logarithmic scale); Figure S4: Geometric mean/SD concentration time profiles of filapixant in plasma after a single oral administration of 60 mg given as a solution (LSF) or a tablet, both in fasted state (linear and semi-logarithmic scale); Figure S5: Geometric mean/SD concentration time profiles of filapixant in plasma per treatment after a single oral administration at doses between 6 and 120 mg given as a solution (liquid service formulation [LSF]), all in fasted state (semi-logarithmic scale); Figure S6: Geometric mean/SD concentration time profiles of filapixant in plasma per treatment after a single oral administration at doses between 250 and 1250 mg administered as a tablet, all in fasted state (semi-logarithmic scale); Table S1: Full list of in- and exclusion criteria; Table S2: Number of subjects with treatment-emergent, study-drug-related AEs by primary system organ class and preferred term; Table S3: Results from the ANOVA analysis for the assessment of dose proportionality of BAY 1902607 in plasma (N = 53); Table S4: Results from the power model for the assessment of dose proportionality of BAY 1902607 in plasma (N = 53).

## Abbreviations

AE	Adverse event
ANOVA	Analysis of variance
ATP	Adenosine triphosphate
CI	Confidence interval
CV	Coefficient of variation
ECG	Electrocardiogram
h	Hour(s)
IC_50_	Concentration for 50% inhibition
LC-MS/MS	High-performance liquid chromatography coupled with tandem mass spectrometry
LLOQ	Lower limit of quantification
LSF	Liquid service formulation
LS means	Least square means
min	Minute
PK	Pharmacokinetics
QC	Quality control
SD	Standard deviation
TEAE	Treatment-emergent adverse event

## Nomenclature

AE,ur	Amount excreted into urine from time zero to infinity
AUC	Area under the concentration–time curve from zero to infinity after single dose
AUC(0 24)	AUC from time zero to 24 h
AUC(0 72)	AUC from time zero to 72 h
AUC(0 tlast)	AUC from time zero to last data point above LLOQ
AUC(0 tlast)/D	AUC from time zero to last data point above LLOQ divided by dose
AUC/D	Area under the concentration–time curve from zero to infinity after single dose divided by dose
AUCnorm	Area under the concentration–time curve from zero to infinity after single dose divided by dose per body weight
AUCu	Area under the concentration–time curve from zero to infinity after single dose of unbound drug
CL/F	Total body clearance of drug calculated after extravascular administration (e.g., apparent oral clearance)
Cmax	Maximum observed drug concentration after single dose administration
Cmax/D	Cmax divided by dose
t1/2	Half-life associated with the terminal slope
tmax	Time to reach Cmax after single dosing
Vz/F	Apparent volume of distribution during terminal phase after extravascular administration
