# Supplementary material for: Safety, Tolerability, and Pharmacokinetics of Filapixant, a Highly Selective P2X3 Receptor Antagonist, in an Ascending-Single-Dose First-in-Human Study
Source: Pharmaceuticals (Basel). 2025 May 20;18(5):758. doi: 10.3390/ph18050758 (PMC12114988; doi:10.3390/ph18050758)
Supplement: Supplementary file 1 [file pharmaceuticals-18-00758-s001.zip › pharmaceuticals-3503018-supplementary.pdf]

Online supplementary appendix

Table S1: Full list of in / exclusion criteria

Inclusion criteria

Persons must fulfill all of the following criteria before being included in the treatment period:

1. Signature of the informed-consent form before any study specific tests or procedures are performed.

2. Confirmation of the subject's health insurance coverage before the first screening visit.

3. Healthy male, according to complete medical history, physical examination, vital signs (blood pressure, pulse rate), 12-lead ECG and clinical laboratory tests.

4. Age: 18 to 45 years (inclusive) at the first screening visit.

5. Body mass index: ≥ 18 kg/m² and ≤ 30 kg/m².

6. Race: White.

7. Smoking of less than 10 cigarettes per day.

8. Agreement by the subject that he and his female partner of childbearing potential will use an accepted method of contraception for the duration of the study, i.e., for the entire period between signing of the informed-consent form and 30 days after the last administration of study drug.

[Note: The parallel application of two of the following methods (mechanical barrier in addition to hormonal contraception) of contraception is considered adequately reliable:

Mechanical barrier (condom for the subject), in addition to hormonal contraception of the female partner (oral contraception with a Pearl index <1, hormone patch, hormone ring, hormone spiral, three-month depot injection or implants), or copper spiral or sterilized (tubal ligation, hysterectomy) female partner.]

9. Ability to understand and follow study-related instructions.

Table S 1: Exclusion criteria

Patients are to be excluded from the study if they display any of the following criteria:

Medical and surgical history

1. Any findings from the medical examination (including medical history, physical examination, vital signs, laboratory tests and ECG) deviating from normal and deemed by the investigator to be of clinical relevance.

2. Relevant diseases within the 4 weeks before the first drug administration.

3. Febrile illness within the week before the first taste test is conducted.

4. Visit to the dentist within the 2 weeks before screening.

5. Xerostomia.

6. Diseases of the oral mucosa.

7. Relevant gastrointestinal diseases.

8. Relevant respiratory diseases.

9. Known severe allergies, non-allergic drug reactions, or multiple drug allergies.

10. Existing chronic diseases requiring medication.

11. History of cardiovascular disease, renal disorders, liver disorders, thyroid disorders or malignant tumors.

12. Known or suspected malignant tumors or carcinoma *in situ* (including history of malignant tumors, with a status after treatment), known or suspected benign tumors of the liver and pituitary (including after treatment).

13. Known liver diseases: existing acute or chronic progressive liver diseases, e.g., disturbances of the bilirubin excretion (Dubin–Johnson or Rotor syndrome); disturbances of the bile secretion and flow (cholestasis); presence or history of liver tumors (benign or malignant). Furthermore, between the subsidence of a viral hepatitis (normalization of liver parameters) and the screening for this study there must have been an interval of at least 6 months.

14. Known relevant kidney diseases (e.g. glomerulonephritis), or renal injury associated with multisystem diseases/disorders (e.g. systemic lupus erythematosus, diabetic nephropathy).

15. Known current hormonal disorders (e.g. thyroid disorders) which require treatment.

16. Known metabolic disorders (e.g. diabetes mellitus, severe hypertriglyceridemia).

17. Known immunological disorders.

18. Known cardiovascular disorders requiring treatment.

19. Migraine with neurological symptoms (complicated migraine).

20. Clinically significant depression (current or in the year before screening).

21. Pancreatitis or a history thereof if associated with severe hypertriglyceridemia.

22. Incompletely cured pre-existing diseases for which it can be assumed that the absorption, distribution, metabolism, elimination and/or effects of the study drugs will not be normal.

23. History of orthostatic hypotension, fainting spells and blackouts.

24. Known hypersensitivity to the study medication(s) including components of the preparation.

25. Known hypersensitivity to components of the American breakfast (dose group 4 only).

26. Medical history of hypogeusia/dysgeusia.

Medication, drug use and special behavioral patterns

27. Regular use of therapeutic or recreational drugs, e.g. carnitine products, anabolics, high-dose vitamins.

28. Suspicion of drug or alcohol abuse.

29. Intake of drugs with a long half-life (>24 hours) within one month before study drug administration.

30. Use of any drugs that might influence the results of the trial; this applies from 14 days before study drug administration and during the entire trial until follow-up, and it includes drugs that might affect the PK (e.g. laxatives, loperamide, metoclopramide, antacids, H_2-receptor antagonists, any broad-spectrum antibiotic, CYP3A4 inducers, CYP3A4 inhibitors).

31. Vegetarian or special diets preventing the subject from eating the standard meals during the study; this includes in particular such diets as might prevent the subject from eating the high-fat high-calorie American breakfast (dose group 4 (60 mg) only) and reluctance/inability on the part of the subject to ingest it.

32. Regular daily consumption of more than 1 L of xanthine-containing beverages, e.g. coffee, tea, cocoa.

33. Donation of blood or plasmapheresis during a period from one month before study drug administration and during the entire study up to 30 days after the last administration of study drug OR up to the subject's last study visit if the post-treatment follow-up period is longer than 30 days.

34. Intake of alcohol within the 48 hours before the study drug administration.

35. Regular daily consumption of more than 500 mL of normal-strength beer or the equivalent quantity of approximately 20 g of alcohol in any other form.

36. Inability to taste at least the second highest concentration of each taste quality using the taste strips.

37. Any drug taken, or any medical procedure conducted, within the two weeks before screening, if the drug/procedure resulted in a relevant decrease in taste sensation.

Electrocardiogram (ECG), blood pressure, heart rate

38. Clinically relevant ECG findings such as a second- or third-degree atrioventricular block, prolongation of the QRS complex over 120 msec or of the QTcB-interval over 450 msec (after at least 10 minutes in a supine position).

39. Systolic blood pressure below 90 mmHg or above 140 mmHg (after at least 10 minutes in a supine position).

40. Diastolic blood pressure below 60 mmHg or above 90 mmHg (after at least 10 minutes in a supine position).

41. Heart rate below 50 beats/min or above 95 beats/min (after at least 10 minutes in a supine position).

Physical examination

42. Clinically relevant findings in the physical examination.

Laboratory examination

43. Clinically relevant deviations of the screened laboratory parameters from their respective reference ranges.

44. Positive result(s) for hepatitis B virus surface antigen (HBsAg), hepatitis C virus antibodies (anti-HCV) or human immune deficiency virus antibodies (anti-HIV 1+2).

45. Positive result in urine drug screening.

46. Subjects with thyroid disorders as evidenced by assessment of clinically relevant thyroid-stimulating hormone (TSH) levels outside the normal reference range at screening.

47. Liver enzymes (ALT, AST, GGT) values above the upper limit of normal at screening.

Other

48. Previous (within the 2 months before study drug administration) or concomitant participation in another clinical study with investigational medicinal product(s).

49. Exclusion periods from other studies.

50. Subject in custody by order of an authority or a court of law.

51. Criteria which in the opinion of the investigator preclude participation for scientific reasons, for reasons of compliance, or for reasons of the subject's safety.

52. Previous assignment to treatment (e.g. randomization) during this study (because allowing previously randomized subjects to be re-included into the study may lead to bias).

53. Scheduled (elective) surgery or planned hospitalization, scheduled to take place after signing the informed consent form and up to 6 weeks after the administration of the study drug.

54. Prolonged immobilization, major surgery; any surgery to the legs or major trauma unless complete remobilization is achieved at least 4 weeks before the first screening examination.

55. Close affiliation with the investigation site, e.g. the subject is a close relative of the investigator or a dependent person, such as an employee or a student at the investigation site).

56. The subject is an employee of Bayer AG or of CRS Clinical Research Services Berlin GmbH.

57. Inability and/or unwillingness to comply with study restrictions.

Table S 2: Number of subjects with treatment-emergent, study drug-related AEs by primary system organ class and preferred term (safety analysis set, N=72)

**Primary SOC**<br>**Preferred term**<br>**MedDRA version 21.0**	**6 mg LSF fasted**<br>**n=6 (100%)**	**15 mg LSF fasted**<br>**n=6 (100%)**	**30 mg LSF fasted**<br>**n=6 (100%)**	**60 mg LSF fasted**<br>**n=6 (100%)**	**120 mg LSF fasted**<br>**n=5 (100%)**	**Placebo LSF fasted**<br>**n=11 (100%)**	**60 mg tablet fed**<br>**n=6 (100%)**	**Placebo tablet fed**<br>**n=2 (100%)**
Number of subjects (%) with at least one AE:	1 (16.7%)	0	2 (33.3%)	1 (16.7%)	2 (40.0%)	2 (18.2%)	0	0
Gastrointestinal disorders	0	0	0	1 (16.7%)	0	0	0	0
Abdominal distension	0	0	0	0	0	0	0	0
Dry mouth	0	0	0	0	0	0	0	0
Feces soft	0	0	0	0	0	0	0	0
Hypoesthesia oral	0	0	0	1 (16.7%)	0	0	0	0
Nausea	0	0	0	0	0	0	0	0
Salivary hypersecretion	0	0	0	0	0	0	0	0
Tongue discomfort	0	0	0	0	0	0	0	0
Vomiting	0	0	0	0	0	0	0	0
Nervous system disorders	1 (16.7%)	0	2 (33.3%)	0	2 (40.0%)	2 (18.2%)	0	0
Ageusia	0	0	0	0	0	0	0	0
Dysgeusia	0	0	2 (33.3%)	0	1 (20.0%)	1 (9.1%)	0	0
Headache	0	0	0	0	1 (20.0%)	1 (9.1%)	0	0
Hypogeusia	0	0	0	0	0	0	0	0
Memory impairment	1 (16.7%)	0	0	0	0	1 (9.1%)	0	0
Vascular disorders	0	0	0	0	0	1 (9.1%)	0	0
Hot flush	0	0	0	0	0	1 (9.1%)	0	0

Source: Table 14.3.1 / 9

Table S2 continued: Number of subjects with treatment-emergent, study drug-related AEs by primary system organ class and preferred term (safety analysis set, N=72) (continued)

**Primary SOC**<br>**Preferred term**<br>**MedDRA version 21.0**	**60 mg tablet fasted**<br>**n=6 (100%)**	**250 mg tablet fasted**<br>**n=6 (100%)**	**500 mg tablet fasted**<br>**n=6 (100%)**	**800 mg tablet fasted**<br>**n=6 (100%)**	**1250 mg tablet fasted**<br>**n=6 (100%)**	**PLC tablet fasted**<br>**n=10 (100%)**	**Total**<br>**n=72 (100%)**
Number of subjects (%) with at least one AE:	0	2 (33.3%)	4 (66.7%)	5 (83.3%)	6 (100.0%)	2 (20.0%)	27 (37.5%)
Gastrointestinal disorders	0	1 (16.7%)	0	2 (33.3%)	6 (100.0%)	1 (10.0%)	11 (15.3%)
Abdominal distension	0	0	0	0	1 (16.7%)	0	1 (1.4%)
Dry mouth	0	0	0	0	2 (33.3%)	0	2 (2.8%)
Feces soft	0	0	0	0	1 (16.7%)	0	1 (1.4%)
Hypoesthesia oral	0	0	0	0	0	1 (10.0%)	2 (2.8%)
Nausea	0	0	0	2 (33.3%)	1 (16.7%)	0	3 (4.2%)
Salivary hypersecretion	0	0	0	1 (16.7%)	2 (33.3%)	0	3 (4.2%)
Tongue discomfort	0	1 (16.7%)	0	0	0	0	1 (1.4%)
Vomiting	0	0	0	0	1 (16.7%)	0	1 (1.4%)
Nervous system disorders	0	2 (33.3%)	4 (66.7%)	5 (83.3%)	6 (100.0%)	1 (10.0%)	25 (34.7%)
Ageusia	0	0	0	0	1 (16.7%)	0	1 (1.4%)
Dysgeusia	0	2 (33.3%)	2 (33.3%)	5 (83.3%)	4 (66.7%)	0	17 (23.6%)
Headache	0	0	1 (16.7%)	2 (33.3%)	2 (33.3%)	1 (10.0%)	8 (11.1%)
Hypogeusia	0	0	3 (50.0%)	3 (50.0%)	5 (83.3%)	0	11 (15.3%)
Memory impairment	0	0	0	0	0	0	2 (2.8%)
Vascular disorders	0	0	0	0	0	0	1 (1.4%)
Hot flush	0	0	0	0	0	0	1 (1.4%)

The column 'Total' counts the subjects over all treatment periods. This is not necessarily the sum of the other columns.

Table S3: Pharmacokinetic parameters of filapixant in plasma after a single oral administration at doses between 6 and 120 mg given as solution (LSF) [geometric mean/%CV (range)].

**Parameter**	**Unit**	**6 mg LSF fasted n=6**	**15 mg LSF fasted n=6**	**30 mg LSF fasted n=6**	**120 mg LSF fasted n=5**
AUC(0-t_{last})	µg·h/L	50.3/26.3 (40.8-83.2)	116/31.3 (77.4-154)	324/33.0 (212-544)	1210/24.2 (863-1660)
AUC(0-24)	µg·h/L	45.5/24.1 (37.6-72.0)	105/29.5 (70.5-135)	281/28.9 (201-466)	1050/19.7 (797-1360)
AUC(0-72)	µg·h/L	52.4/25.4 (43.3-85.4)	119/31.4 (78.7-159)	324/32.5 (214-544)	1210/23.8 (863-1640)
AUC(0-t_{last})/D	10^{-3}·h/L	8.38/26.3 (6.81-13.9)	7.75/31.3 (5.16-10.3)	10.8/33.0 (7.08-18.1)	10.1/24.2 (7.19-13.8)
AUC_{norm}	kg·h/L	0.772/28.1 (0.579-1.20)	0.627/26.3 (0.449-0.848)	0.849/26.5 (0.588-1.17)	0.786/35.1 (0.535-1.23)
CL/F	L/h	114/25.5 (69.8-138)	126/31.6 (93.5-190)	91.7/32.9 (54.7-140)	98.8/24.3 (72.1-139)
V_Z/F	L	1590/31.2 (1010-2460)	1720/24.5 (1400-2680)	1490/29.4 (871-2050)	1610/18.1 (1420-2180)
AUC_u	µg·h/L	10.5/25.5 (8.65-17.1)	23.7/31.6 (15.7-31.9)	65.1/32.9 (42.6-109)	242/24.3 (172-331)

Pharmacokinetic parameters of filapixant in plasma after a single oral administration of 60 mg given as solution (LSF) or tablet in fasted state or as tablet concomitantly with food [geometric mean/%CV (range)]

Parameter	Unit	60 mg LSF fasted n=6	60 mg tablet fasted n=6	60 mg tablet fed n=6
AUC(0-t_{last})	µg·h/L	492/39.5 (288-833)	420/43.5 (204-645)	629/38.2 (347-943)
AUC(0-24)	µg·h/L	425/36.4 (271-709)	360/39.5 (191-560)	514/33.7 (321-781)
AUC(0-72)	µg·h/L	491/39.2 (290-834)	419/42.9 (206-645)	624/37.4 (351-937)
AUC(0-t_{last})/D	10^{-3}·h/L	8.21/39.5 (4.80-13.9)	7.01/43.5 (3.41-10.8)	10.5/38.2 (5.79-15.7)
AUC_{norm}	kg·h/L	0.664/31.9 (0.456-0.917)	0.567/36.2 (0.324-0.820)	0.847/32.4 (0.552-1.29)
CL/F	L/h	121/39.4 (71.4-207)	142/43.4 (92.5-291)	94.8/38.0 (63.5-171)
V_Z/F	L	2050/34.2 (1180-3120)	2350/29.0 (1410-3140)	1660/28.3 (1010-2210)
AUC_u	µg·h/L	98.8/39.4 (57.7-167)	84.3/43.4 (41.0-129)	126/38.0 (69.9-188)

Pharmacokinetic parameters of filapixant in plasma after a single oral administration at doses between 250 and 1250 mg given as tablet (geometric mean/%CV (range))

Parameter	Unit	250 mg tablet fasted n=6	500 mg tablet fasted n=6	800 mg tablet fasted n=6	1250 mg tablet fasted n=6
AUC(0-t_{last})	µg·h/L	2050/24.5 (1670-3200)	4760/7.86 (4260-5350)	6040/39.0 (4000-11600)	14600/35.3 (8740-23300)
AUC(0-24)	µg·h/L	1640/21.9 (1350-2460)	3840/10.9 (3360-4610)	4780/45.2 (2960-9900)	11300/36.2 (6970-18300)
AUC(0-72)	µg·h/L	2020/24.1 (1660-3140)	4680/8.30 (4160-5320)	5980/39.3 (3960-11600)	14300/35.1 (8620-22900)
AUC(0-t_{last})/D	10^{-3}·h/L	8.18/24.5 (6.70-12.8)	9.52/7.86 (8.52-10.7)	7.55/39.0 (5.00-14.5)	11.7/35.3 (6.99-18.6)
AUC_{norm}	kg·h/L	0.646/23.1 (0.496-0.894)	0.807/13.1 (0.635-0.907)	0.623/31.2 (0.406-1.02)	0.974/34.1 (0.594-1.50)
CL/F	L/h	122/24.5 (78.0-149)	105/7.88 (93.2-117)	132/39.0 (68.8-200)	85.4/35.4 (53.7-143)
V_Z/F	L	2500/26.5 (1650-3350)	2280/26.9 (1610-3010)	2450/52.3 (1040-3740)	1770/33.5 (1150-2740)
AUC_u	µg·h/L	408/24.5 (334-638)	948/7.88 (849-1070)	1200/39.0 (797-2310)	2910/35.4 (1740-4630)

a Median (range).

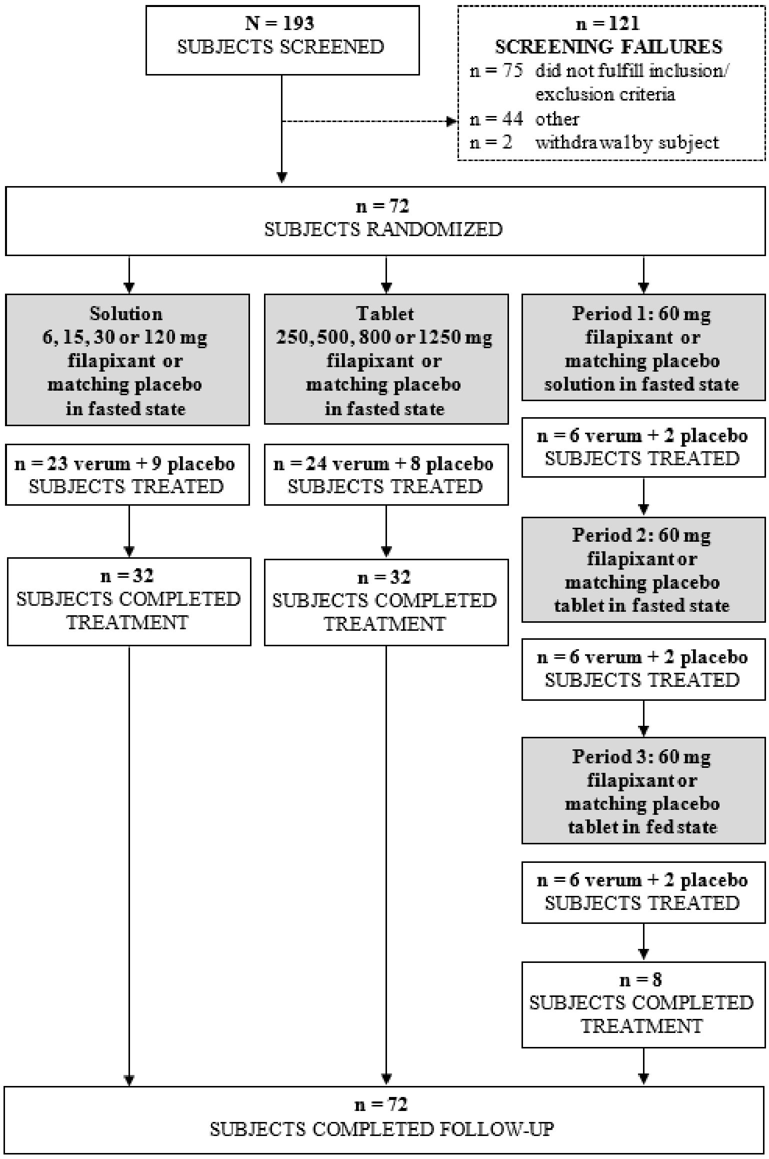

Figure S 1: Subject disposition

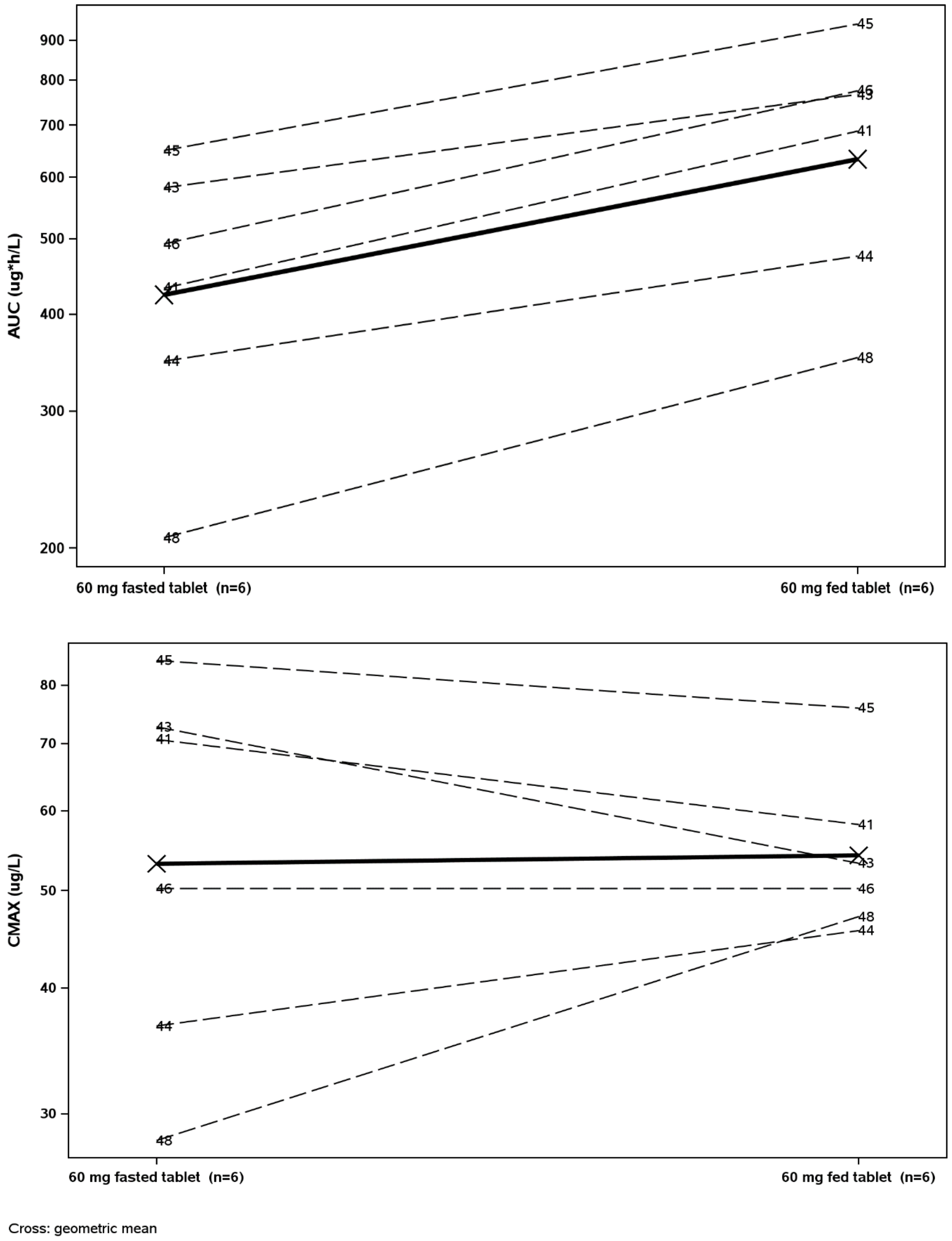

Figure S 2: Stick plot for AUC (µg·h/L) and Cmax (µg/L) of filapixant in plasma by treatment - investigation of food effect (logarithmic scale)

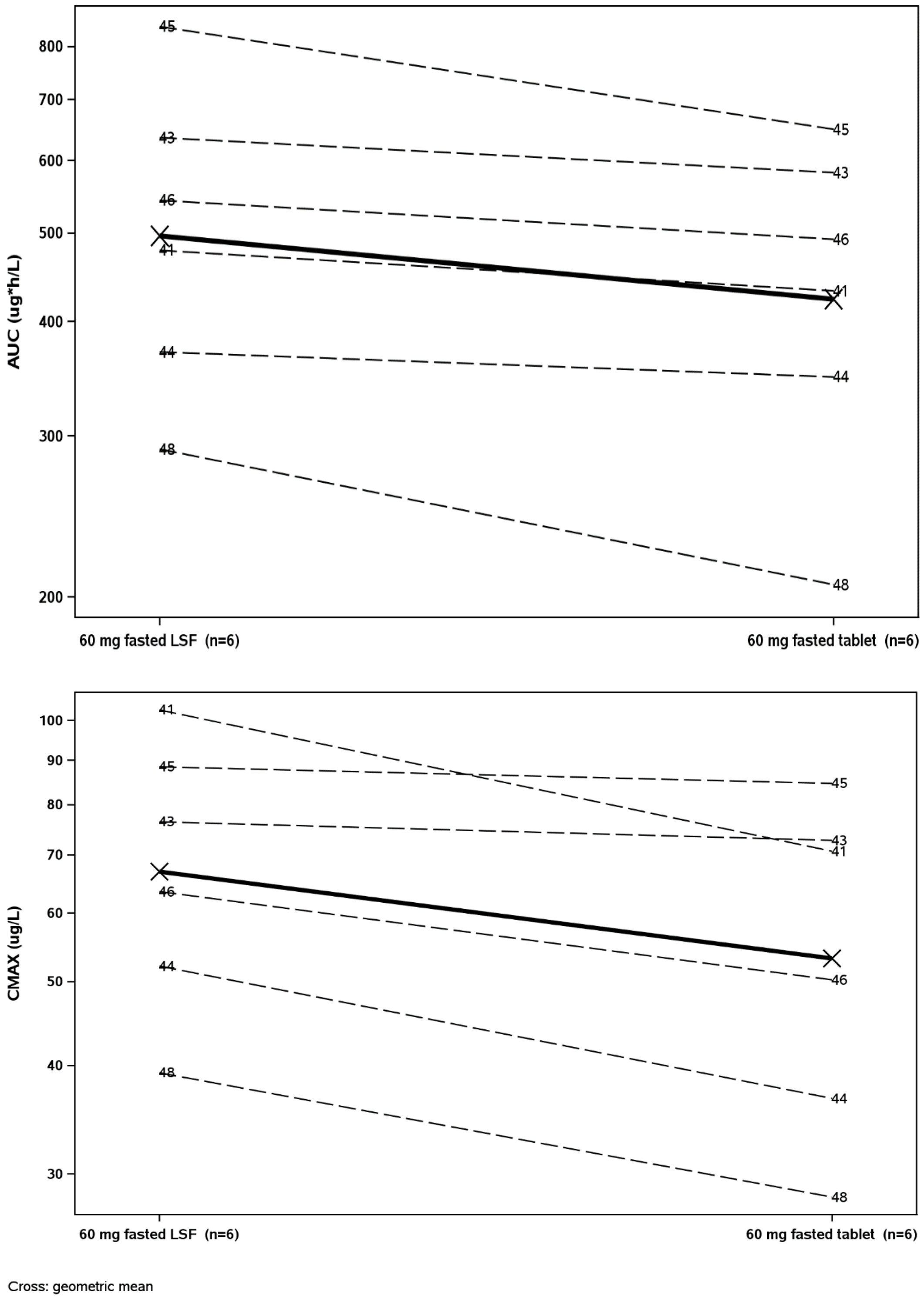

Figure S 3: Stick plots for AUC (µg·h/L) and Cmax (µg/L) of filapixant in plasma by treatment - investigation of relative bioavailability (logarithmic scale)

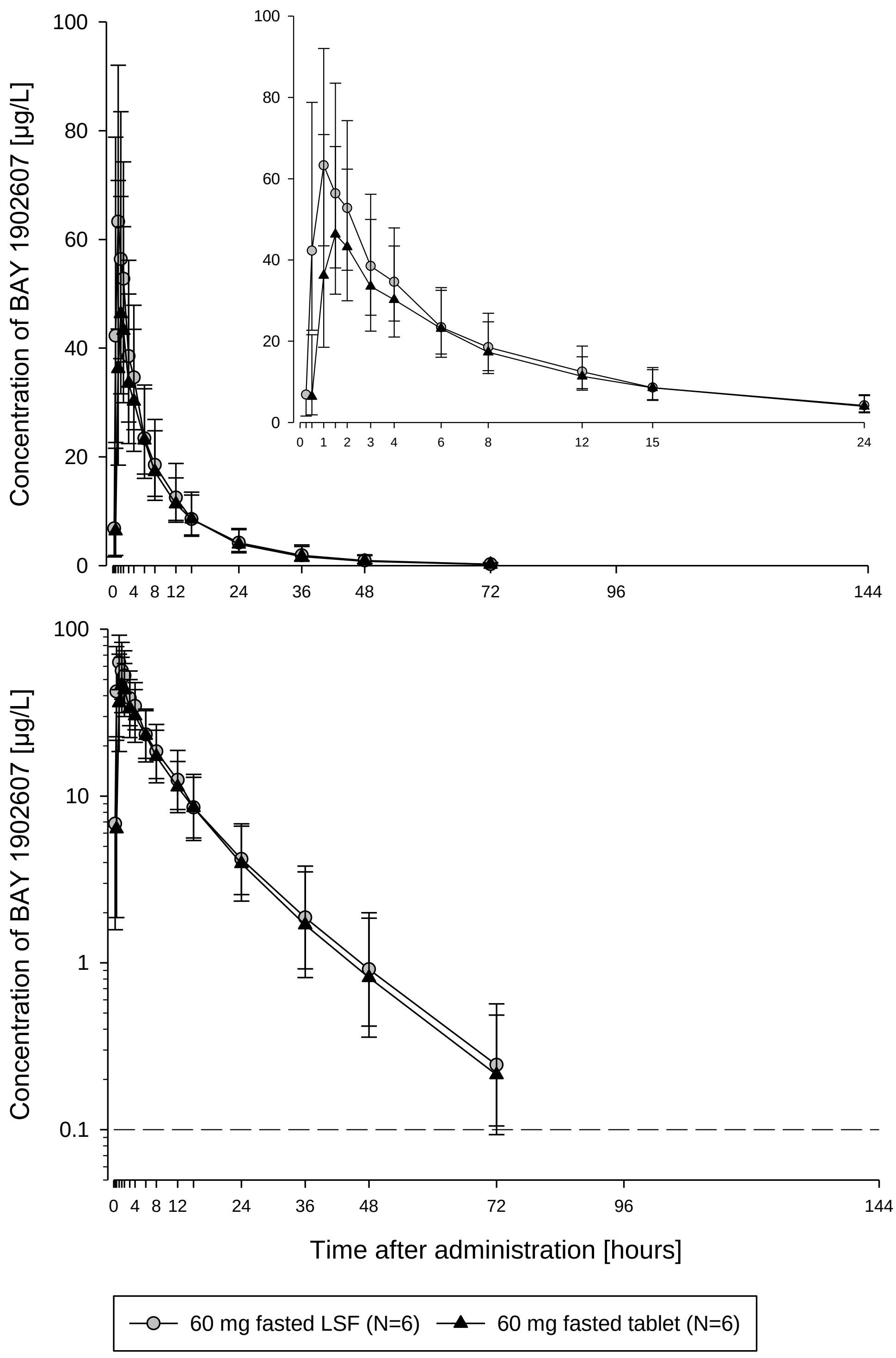

LLOQ = 0.1 µg/L

Figure S 4: Geometric mean/SD concentration time profiles of filapixant in plasma after a single oral administration of 60 mg given as LSF or tablet, both in fasted state (linear and semi-logarithmic scale)

Table S4: Results from the ANOVA analysis for the assessment of dose-proportionality of BAY 1902607 in plasma (N=53)

Parameter	Unit	Group	Point estimate LS-Means	90% CI	Pr > F
AUC/D	h/L	6 mg LSF fasted	0.0088	[0.0071; 0.0109]	0.3661
		15 mg LSF fasted	0.0080	[0.0064; 0.0098]	
		30 mg LSF fasted	0.0109	[0.0088; 0.0135]	
		60 mg LSF fasted	0.0083	[0.0067; 0.0102]	
		120 mg LSF fasted	0.0101	[0.0080; 0.0128]	
		250 mg tablet fasted	0.0082	[0.0067; 0.0100]	0.0703
		500 mg tablet fasted	0.0095	[0.0078; 0.0116]	
		800 mg tablet fasted	0.0076	[0.0062; 0.0092]	
		1250 mg tablet fasted	0.0117	[0.0096; 0.0143]	
		6 mg LSF fasted	0.0088	[0.0072; 0.0108]	0.1688
		15 mg LSF fasted	0.0080	[0.0065; 0.0097]	
		30 mg LSF fasted	0.0109	[0.0089; 0.0134]	
		60 mg LSF fasted	0.0083	[0.0067; 0.0101]	
		120 mg LSF fasted	0.0101	[0.0081; 0.0126]	
		250 mg tablet fasted	0.0082	[0.0067; 0.0100]	
		500 mg tablet fasted	0.0095	[0.0078; 0.0117]	
		800 mg tablet fasted	0.0076	[0.0062; 0.0093]	
		1250 mg tablet fasted	0.0117	[0.0096; 0.0144]	
C_{max}/D	/L	6 mg LSF fasted	0.0010	[0.0008; 0.0013]	0.0575
		15 mg LSF fasted	0.0010	[0.0008; 0.0012]	
		30 mg LSF fasted	0.0016	[0.0013; 0.0020]	
		60 mg LSF fasted	0.0011	[0.0009; 0.0014]	
		120 mg LSF fasted	0.0014	[0.0011; 0.0018]	
		250 mg tablet fasted	0.0009	[0.0007; 0.0012]	0.1241
		500 mg tablet fasted	0.0011	[0.0008; 0.0014]	
		800 mg tablet fasted	0.0007	[0.0005; 0.0009]	
		1250 mg tablet fasted	0.0011	[0.0008; 0.0014]	
		6 mg LSF fasted	0.0010	[0.0008; 0.0013]	0.0071
		15 mg LSF fasted	0.0010	[0.0008; 0.0013]	
		30 mg LSF fasted	0.0016	[0.0013; 0.0021]	
		60 mg LSF fasted	0.0011	[0.0009; 0.0014]	
		120 mg LSF fasted	0.0014	[0.0011; 0.0018]	
		250 mg tablet fasted	0.0009	[0.0007; 0.0011]	
		500 mg tablet fasted	0.0011	[0.0008; 0.0014]	
		800 mg tablet fasted	0.0007	[0.0005; 0.0008]	
		1250 mg tablet fasted	0.0011	[0.0009; 0.0014]	

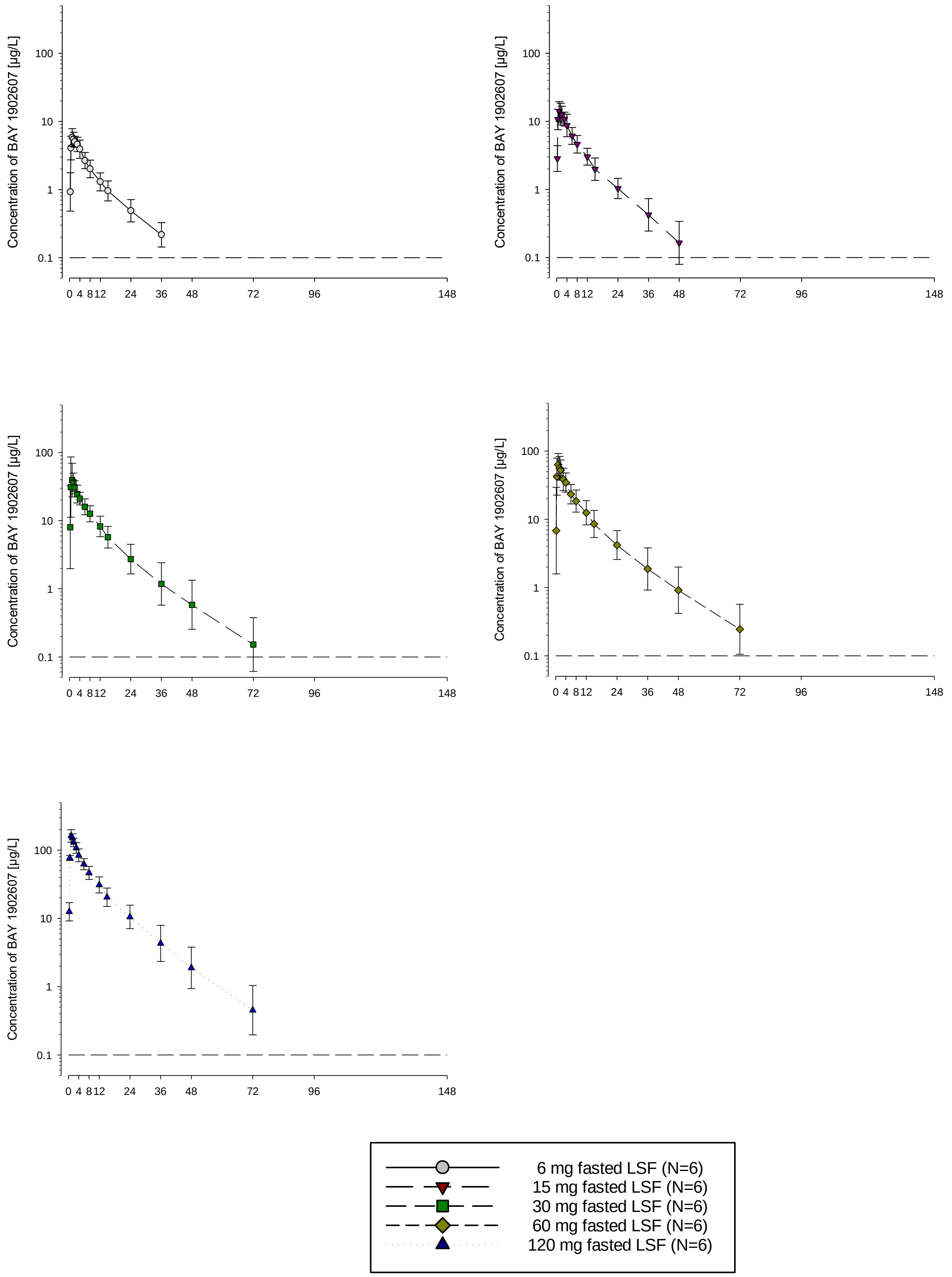

Figure S 5: Geometric mean/SD concentration time profiles of filapixant in plasma per treatment after a single oral administration at doses between 6 and 120 mg given as solution (liquid service formulation [LSF]), all in fasted state (semi-logarithmic scale)

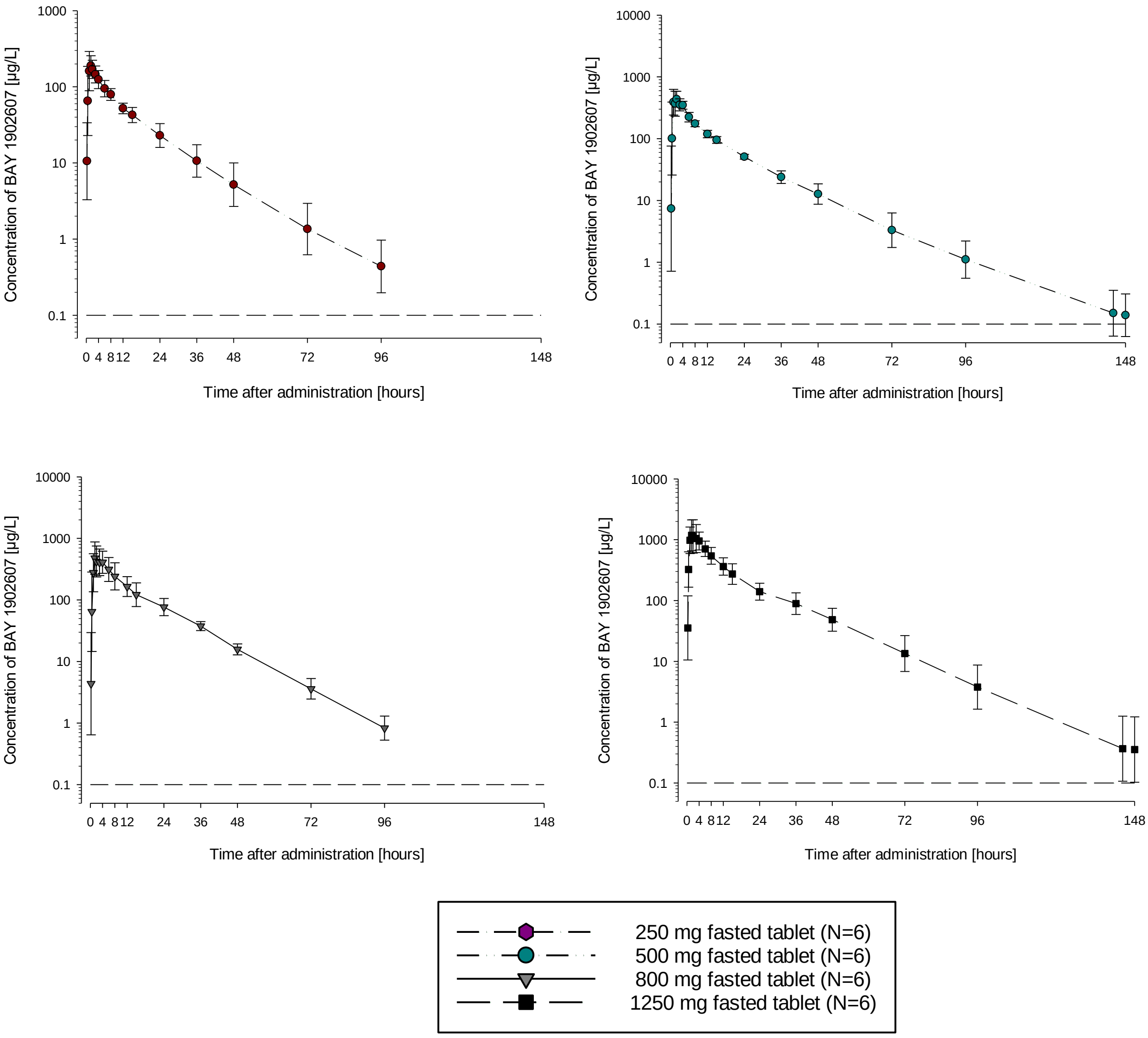

Figure S 6: Geometric mean/SD concentration time profiles of filapixant in plasma per treatment after a single oral administration at doses between 250 and 1250 mg administered as tablet, all in fasted state (semi-logarithmic scale)
